# EHMTI-0358. Improved chronic migraine after DTMS

**DOI:** 10.1186/1129-2377-15-S1-M10

**Published:** 2014-09-18

**Authors:** P Scatena, G Sette, C Rapinesi, GD Kotzalidis, VR Ferri, S Di Pietro, RN Raccah, S Ferracuti, F Orzi, P Girardi

**Affiliations:** 1 NESMOS (Neuroscience Mental Health and Sensory Organs), "Sapienza" Università di Roma, Roma, Italy; 2 Brainsway-Italy, "Sapienza" Università di Roma, Roma, Italy

## Background

The prevalence of chronic migraine (CM) in the general population is around 2%. CM occurs with impaired quality-of-life and frequent medication overuse. Deep transcranial magnetic stimulation (dTMS) of the dorsolateral prefrontal cortex (DLPFC) transiently suppresses central pain perception through reduced functional connectivity between mid-brain and medial thalamus.

## Aim

To assess pain reduction in CM using high frequency rTMS over the left DLPFC.

## Method

Fourteen patients with ICHD-32 CM were randomised to 12 dTMS sessions, delivered on alternate days over bilateral DLPFC with left prevalence (N=7; 6 women, 1 man; mean age 45 years) or to treatment as usual (TAU, with anti-migraine agents). All had severe headaches for ≥15 days/month in the last three months, and did not respond to ≥3 preventive medications and to drug overuse treatment. Outcome measures were attack frequency, headache index, and number of medications in the month before (baseline), during treatment, and one month later.

## Results

Patients treated with dTMS, compared to TAU and baseline, had reduced pain intensity, frequency of attacks, and analgesic overuse, during treatment and one month later.

## Conclusion

dTMS presumably improved DLPFC function, thus allowing better executive abilities. This may have enhanced salience-related brain activity, redirecting or diverting attention through the hippocampus, the cingulate cortex, or other pain matrix structures. Results are compatible with improved brain control over pain sensations. High-frequency dTMS over bilateral DLPFC improved CM, supporting a role for DLPFC in pain control.

No conflict of interest.

## References

[B1] Manzoni Curr Pain Headache Rep 2011 15 170 6 10.1007/s11916-011-0188-0 21365366

